# Supplementary material for: Girdling increases branch capacity to rehydrate in Juniperus thurifera and drought hampers bimodal growth
Source: Quant Plant Biol. 2026 Jan 26;7:e3. doi: 10.1017/qpb.2026.10037 (PMC12930207; doi:10.1017/qpb.2026.10037)
Supplement: Camarero et al. supplementary material [file S263288282610037Xsup001.docx]

**Supporting Information**

| (a) |
| --- |
|  |
| (b) |
|  |

**Figure S1.** (a) Summed precipitation during the hydrological year (from previous October to current September), and (b) weekly values of the 6-month-long SPEI values (SPEI6). Both plots show the dry conditions experienced in the year 2022 (vertical red lines).

**Figure S2.** Cross-section showing several latewood IADFs (yellow arrows) along a *J. thurifera* tree ring. IADFs are characterized by tracheids with wide lumen formed within the latewood.

**Figure S3.** Stem dendrometer records of radial increment obtained at hourly resolution for wet (2020) and dry (2022) years. Values are means ± SD.

**
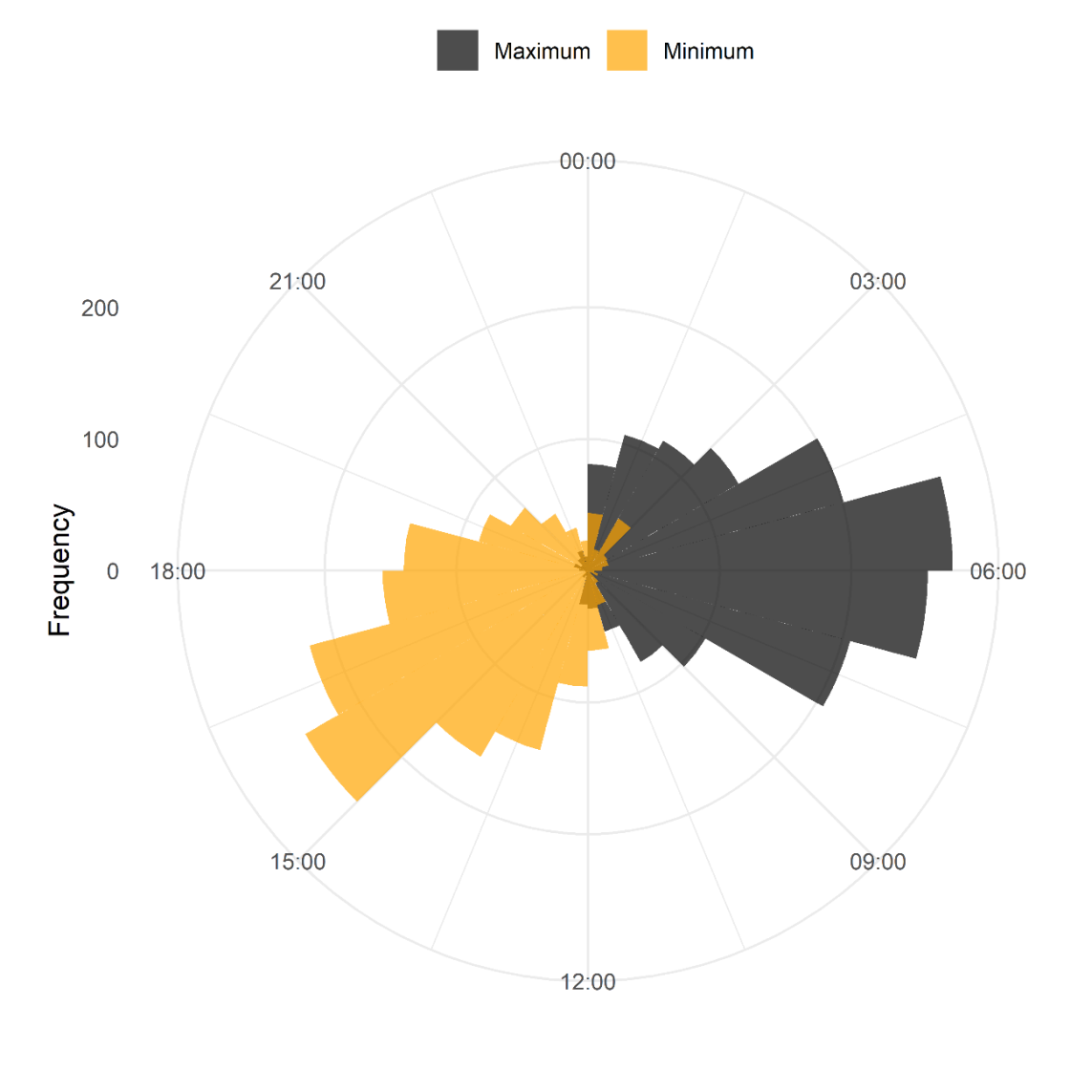
**

**Figure S4.** Time of maximum (black sections) and minimum (yellow sections) daily diameter in *J. thurifera* branches. This circular histogram suggests that daily diameter extremes are driven by transpiration dynamics.


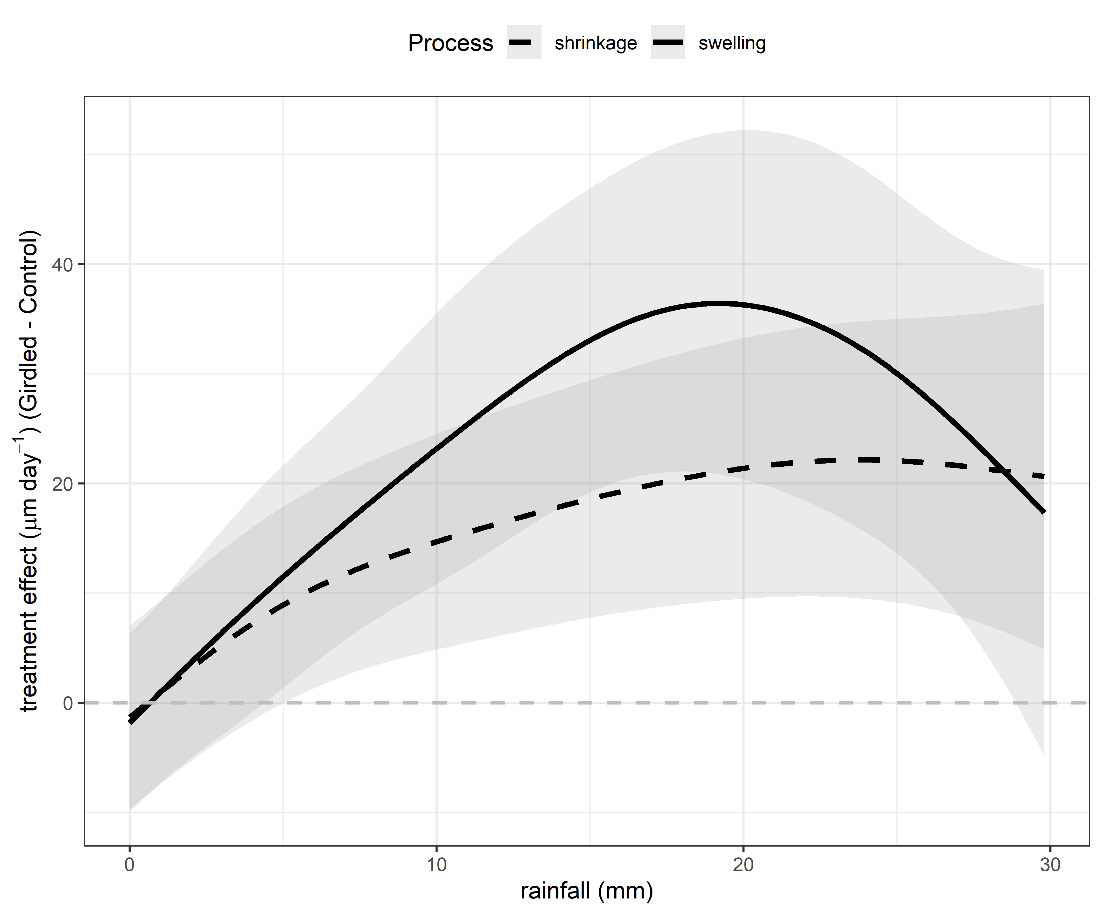


**Figure S5.** Treatment effect (as the difference between girdled and control) on branch shrinkage (continuous line) and swelling (dashed line) across the rainfall gradient and based on a non-linear model. Lines and shaded ribbons show mean ± 95 % confidence intervals.
